# Comparative Chloroplast Genomics of *Fritillaria* (Liliaceae), Inferences for Phylogenetic Relationships between *Fritillaria* and *Lilium* and Plastome Evolution

**DOI:** 10.3390/plants9020133

**Published:** 2020-01-21

**Authors:** Jiao Huang, Yan Yu, Yan-Mei Liu, Deng-Feng Xie, Xing-Jin He, Song-Dong Zhou

**Affiliations:** 1 Key Laboratory of Bio-Resources and Eco-Environment of Ministry of Education, College of Life Science, Sichuan University, Chengdu 610065, Sichuan, China; Fritillaria123@sina.com (J.H.); yyu@scu.edu.cn (Y.Y.); 15208198824@163.com (Y.-M.L.); df_xie2017@163.com (D.-F.X.); 2 College of Life Science, Leshan Normal University, Leshan 614000, Sichuan, China

**Keywords:** *Fritillaria*, Liliaceae, plastome genome, genome structure, phylogenomics

## Abstract

*Fritillaria* is a genus that has important medicinal and horticultural values. The study involved the most comprehensive chloroplast genome samples referring to Old and New World clades of *Fritillaria* for marker selection and phylogenetic studies. We reported and compared eleven newly sequenced whole-plastome sequences of *Fritillaria* which proved highly similar in overall size (151,652–152,434 bp), genome structure, gene content, and order. Comparing them with other species of Liliales (6 out of 10 families) indicated the same similarity but showed some structural variations due to the contraction or expansion of the inverted repeat (IR) regions. A/T mononucleotides, palindromic, and forward repeats were the most common types. Six hypervariable regions (*rps16*-*trnQ, rbcL*-*accD, accD*-*psaI, psaJ*-*rpl33, petD*-*rpoA*, and *rpl32*-*trnL*) were discovered based on 26 *Fritillaria* whole-plastomes to be potential molecular markers. Based on the plastome data that were collected from 26 *Fritillaria* and 21 *Lilium* species, a phylogenomic study was carried out with three *Cardiocrinum* species as outgroups. *Fritillaria* was sister to *Lilium* with a high support value, and the interspecies relationships within subgenus *Fritillaria* were resolved very well. The six hypervariable regions can be used as candidate DNA barcodes of *Fritillaria* and the phylogenomic framework can guide extensive genomic sampling for further phylogenetic analyses.

## 1. Introduction

Plastid genome of angiosperm have a typical quadripartite structure with a pair of inverted repeat (IR) regions separated by a small single-copy (SSC) region and a large single-copy (LSC) region [1,2]. In contrast to mitochondrial and nuclear genomes, plastid genome is highly conserved and generally contains 110–130 distinct genes, ranging in size from 120–160 kb [3]. Although plastid genomes are reported as a highly conserved sequence and structure in most angiosperms, they have been showed considerable variation in many taxa [2]. These structural variations are always involved in the contraction or extension of the IR region [1], appearance of large inversions or deletions [4,5], and genes gain or loss [2,6]. Some hotspot regions with single nucleotide polymorphisms could be identified, which may be used for species identification in terms of enough information [7]. Due to low rates of nucleotide substitutions, lack of recombination, and uniparental inheritance, many plastid DNA sequences have been used for inferring plant phylogenies and population genetic analyses [8,9], such as *matK*, *rbcL*, and *trnH*-*psbA* [10].

Currently, whole chloroplast genomes have been increasingly used for phylogenetic analyses and inferring phylogeographic histories, as the advent and fast development of next-generation sequencing (NGS) technologies. They can provide plenty of variable sites among their entire size for phylogenetic analyses [3]. Thus, whole chloroplast genomes show the potential for resolving evolutionary relationships and have been employed to generate highly resolved phylogenies and genetic diversity, especially in the unresolved relationship of some complex taxa or at low taxonomic levels [2,11,12,13]. Because different regions of the whole chloroplast genomes differed with their evolutionary rates, partitioning the genome by regions or genes might be preferable for phylogenomic analysis [3]. The concatenated coding genes were most widely used for plastome phylogenomics until now [14,15].

The genus *Fritillaria* L. (Liliaceae) contains 130 to 140 species which are mostly distributed in the temperate regions of the Northern Hemisphere. The centers of speciation and diversity of the genus may be in the Qinghai-Tiber Plateau (QTP), Irano-Turanian region, and the Mediterranean Basin [16,17]. Bulbs of *Fritillaria* have long been used as herbs in Asian countries, especially in traditional Chinese medicine, which are called “Bei-mu” in Chinese. Bulbs of *Fritillaria* are important medicine materials, which have been used in relieving cough, clearing heat, eliminating phlegm, analgesic, and detoxifying, and have even been used in treating cancers [18,19]. As the chemical compounds and pharmacological effects greatly differ among *Fritillaria* species, it is crucial to accurately identify them based on morphological and molecular data [18]. Different barcoding such as plastid DNA (*matK*, *rpl16*, *trnL*-*trnF*) and nuclear DNA internal transcribed spacer (ITS), single nucleotide polymorphisms (SNPs), and genomic inter-simple sequence repeats (ISSRs) have been performed for identifying *Fritillaria* species [18,20]. According to previous classification analysis, *Fritillaria* comprises eight subgenera, which are further confirmed monophyletic excepting the subgenus *Fritillaria* based on molecular phylogenetic analyses [17,21]. However, our previous study (using three cpDNA markers) [17] detected that the genus *Fritillaria* was not a monophyly and divided into the New World and Old World clades, and the species of *Lilium* were nested within *Fritillaria* with moderate support, although the ITS data revealed a poor monophyly of *Fritillaria*. Day et al. attempted to adopt low-copy nuclear gene regions as barcoding, but none of the target regions experimented could be amplified across all species tested [16]. These uncertainties about phylogenetic relationships between the genus *Fritillaria* and its neighbors are most likely due to few genetic barcodings used in previous studies, even though confounding influences such as hybridization in the evolutionary processes cannot be excluded. So far, a total of fifteen plastid genomes of genus *Fritillaria* have been sequenced and are available on GenBank. These comparative analyses with different *Fritillaria* plastid genomes have provided a basic understanding of the plastid genome characteristics of this genus [22,23,24,25].

In this study, plastomes of 11 *Fritillaria* species were sequenced, and a genomic comparative analysis was performed combined with other 15 *Fritillaria* and 21 *Lilium* plastomes available from Genbank. The specific purposes of this study were (1) to compare and analyze the plastome structural of the eleven *Fritillaria* species; (2) to identify highly divergent regions of all 26 *Fritillaria* plastomes; and (3) to resolve the evolutionary relationships between genus *Fritillaria* and *Lilium*. Overall, this study would be helpful to further understand the plastome evolution and phylogeneny of *Fritillaria* species.

## 2. Results 

### 2.1. Plastome Features of Fritillaria and Comparison with Other Liliales Taxa

**Figure 1:**
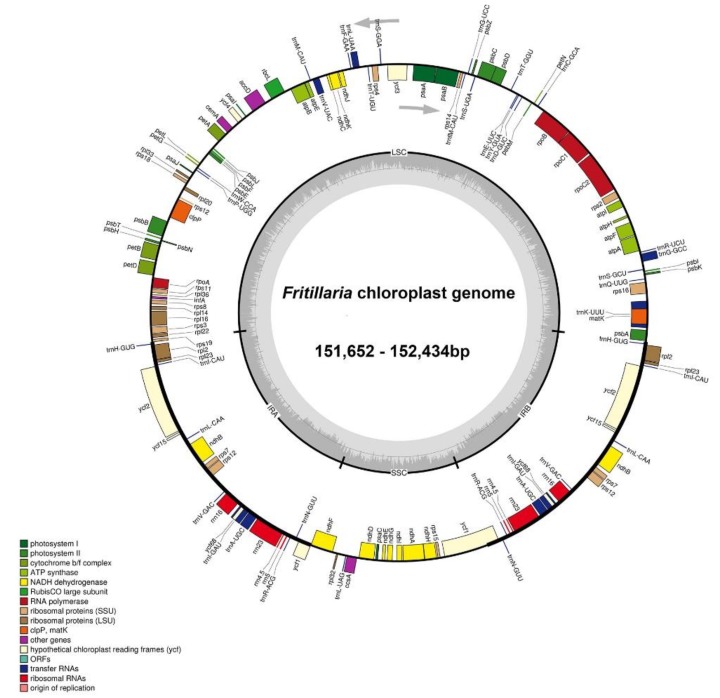
Circular gene map of *Fritillaria* species (*Fritillaria anhuiensis*, *F. crassicaulis*, *F. dajinensis*, *F. davidii*, *F. delavayi*, *F. maximowiczii*, *F. monantha*, *F. przewalskii*, *F. sichuanica*, *F. unibracteata*, and *F. yuzhongensis*) chloroplast genomes. The genes inside and outside of the circle are transcribed in clockwise and counterclockwise directions, respectively. Genes belonging to different functional groups are shown in different colors. The dashed darker gray area in the inner circle indicates genome GC content, while the lighter gray area shows AT content. IR = inverted repeat; SSC = small single copy; LSC = large single copy.

A total of 1.02–2.24 G clean base of the each *Fritillaria* species was collected and the plastomes size ranged from 151,652 bp (*Fritillaria yuzhongensis*) to 152,434 bp (*Fritillaria maximowiczii*) (Table 1). All these plastomes showed the typical quadripartite structure akin to other seed plants, consisting of a pair of IRs (26,232–26,574 bp) separated by the SSC regions (17,310–17,684 bp) and the LSC (81,424–81,976 bp). The whole GC content of these 11 plastomes was very similar (36.9–37.1%). The eleven *Fritillaria* plastid genomes contained about 131–136 predicted functional genes, including 85–90 protein-coding genes, 38 transfer RNA (tRNA) genes, and 8 ribosomal RNA (rRNA) genes (Figure 1, Table 1 and Table S1). Six to eight protein-coding genes, eight tRNA genes, and all four rRNA genes were duplicated in the IR regions. Among these genes, two CDS (*clpP* and *ycf3*) possessed two introns, while ten CDS genes and six tRNA genes contained a single intron (Table S1). The gene *rps12* was a trans-spliced gene with the 5’ end exon located in the LSC region and the 3’ exon and intron located in the IR regions. Four pseudogenes (*ψinfA*, *ψycf1*, *ψycf15*, and *ψycf68*) were identified from five *Fritillaria* plastomes (*F. crassicaulis*, *F. dajinensis*, *F. delavayi*, *F. sichuanica*, *F. unibracteata*), and the *ψycf15* and *ψycf68* were duplicated and located in IRA and IRB regions, respectively. The *rps19* gene at the LSC-IR_A_ border in *F. maximowiczii* was also identified as a pseudogene. In addition, the *F. przewalskii* and *F. yuzhongensis* plastid genome contained two pseudogenes (*ψinfA* and *ψycf1*). The genes *ycf68*, *ycf15*, and *infA* contained several internal stop codons and indicated that they may be identified as pseudogenes [9,22]. Pseudogene for *ycf1* was found at the SSC-IR_B_ junctions and has lost its protein-coding ability because of incomplete gene duplication. The similar phenomenon was also observed in the *rps19* gene at the LSC-IR_A_ border.

**Figure 2:**
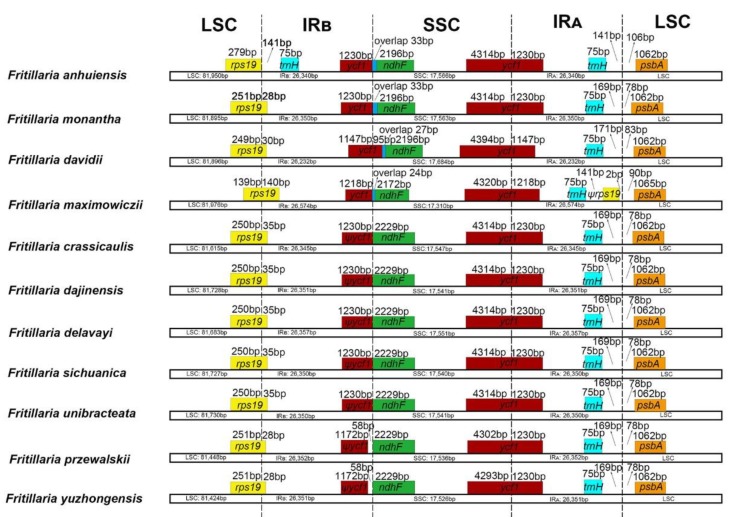
Comparison of LSC, IRs, and SSC junction positions among eleven *Fritillaria* chloroplast genomes.

The IR/SC junction regions with full annotations were compared and had the almost same relative positions among the eleven newly sequencing *Fritillaria* chloroplast genomes (Figure 2). Except for *F. anhuiensis*, all the LSC-IR_B_ junctions were located within the *rps19* gene, resulting in the IR_B_ region expanded by a part (28–140 bp) toward the *rps19* gene. In *F. anhuiensis*, the junction was located at the intergenic spacer region (IGS) between *rps*19 and *trnH-GUG*. The SSC-IR_A_ borders in the eleven plastomes were located in the *ycf1* gene and part of this gene was duplicated from 1147–1230 bp in the IR_B_. In addition, the *ycf1* gene in four species (*F. anhuiensis*, *F. monantha*, *F. davidii*, and *F. maximowiczii*) extended 24–122 bp into the SSC region, which also had a 27–33 bp overlap with *ndhF*. In five species (*F. crassicaulis*, *F. dajinensis*, *F. delevayi*, *F. sichuanica*, *F. unibracteata*), the SSC-IR_B_ boundary positions were located at the junction between the pseudogene *ψycf1* and *ndhF*. However, for the other two species (*F. przewalskii* and *F. yuzhongensis*), the distance between *ψycf1* and *ndhF* varied from 58 bp (Figure 2). The LSC-IR_A_ border was located at the IGS between *trnH-GUG* and *psbA* in all but one species (*F. maximowiczii*). In *F. maximowiczii*, the junction was located in the ψ*rps19*-*psbA* spacer.

Comparison of the 11 newly sequencing *Fritillaria* plastomes with 10 other species of Liliales showed obvious expansion and contraction of the IRs in several families (Figure S1). The LSC-IR_B_ boundary was located in the *rps19* gene in most Liliales species. However, this boundary shifted into the IGS between the *rpl22* and *rps19* genes in Campynemataceae and it expanded a part toward the *rpl22* gene in Smilacaceae. It further shifted into a part of *rps3* in Melanthiaceae (*Paris*). Long *ψycf1* fragment located at the IR_B_ varied from 2555 to 992 bp, and the IR has expanded ~1.0–2.0 kb at the SSC-IR_S_ border in Colchicaceae and Campynemataceae compared to the most Liliales species (see Table S2). Meanwhile, the IR region had a relative contraction in Melanthiaceae (*Veratrum*) (Figure S1). It is also notable that the SSC region of Campynemataceae had a different orientation (inversion) relative to the other Liliales species.

### 2.2. Comparative Genomic Analysis and Divergence Hotspot Regions

**Figure 3:**
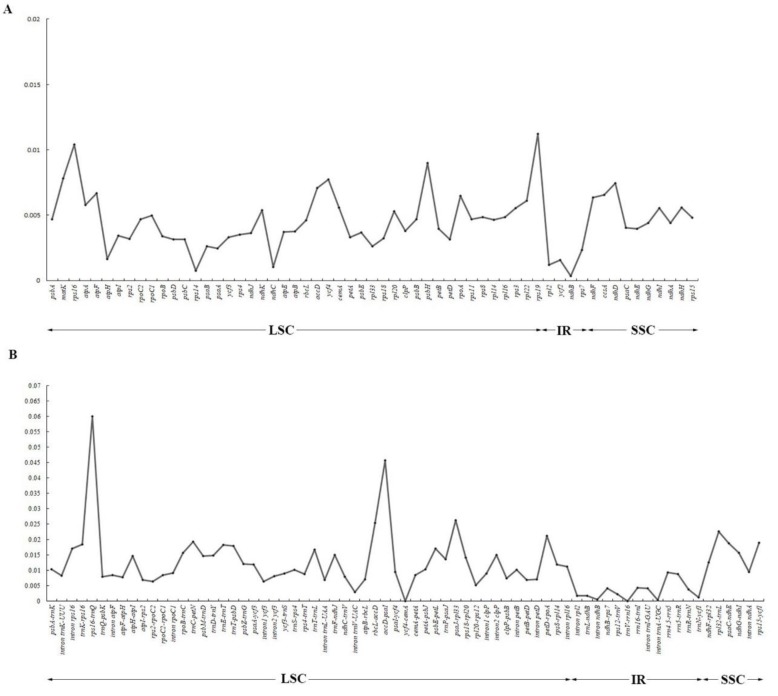
Comparison of nucleotide variability (Pi) values in 26 *Fritillaria* plastomes. (**A**) Pi values among protein-coding genes (CDS); (**B**) Pi values among intergenic spacer (IGS) regions.

We investigated the comprehensive sequence divergence of the 11 newly sequencing *Fritillaria* plastid genomes using mVISTA with *F. cirrhosa* as the reference. The aligned sequences revealed high sequence similarities, and several regions of high sequence length polymorphism were revealed (Figure S2). As expected, IR regions exhibited comparatively fewer sequence divergence than LSC and SSC regions. We identified 130 regions (18 introns, 53 intergenic spacers, and 59 coding regions) with more than 200 bp in length from the 26 *Fritillaria* plastid genomes. Of the 59 protein-coding regions (CDS), nucleotide variability (Pi) for each locus ranged from 0.00036 *(ndhB*) to 0.0112 (*rps19*) and the average of 0.00453. Thereby, seven regions (*matK*, *rps16*, *accD*, *ycf4*, *rps19*, *psbH*, *ndhD*) had remarkably high values (Pi > 0.007; Figure 3A and Table S3). For the 71 noncoding (intergenic spacer and intron) regions, Pi values ranged from 0.0000 (*ycf4*-*cemA* and *trnV*-*rrn16*) to 0.05996 (*rps16*-*trnQ*) and the average of 0.01163. Six of those regions also exhibited considerable high values (Pi > 0.02; i.e., *rps16*-*trnQ, rbcL*-*accD, accD*-*psaI, psaJ*-*rpl33, petD*-*rpoA*, and *rpl32*-*trnL*; see Figure 3B and Table S4). The results proved that the IR regions had more sequence conservation than the SC regions, and the average value of Pi in the non-coding regions was more than twice as much as in the coding regions. Specifically, *rbcL*-*accD*, *accD*-*psaI*, and *rpl32*-*trnL* had the highest resolution and similar topology according to the NJ trees. These six divergence hotspot regions in the noncoding regions should provide a useful resource for future phylogenetic and phylogeographic analyses as well as species identification of genus *Fritillaria*.

### 2.3. SSRs Analysis and Repeat Sequences

**Figure 4:**
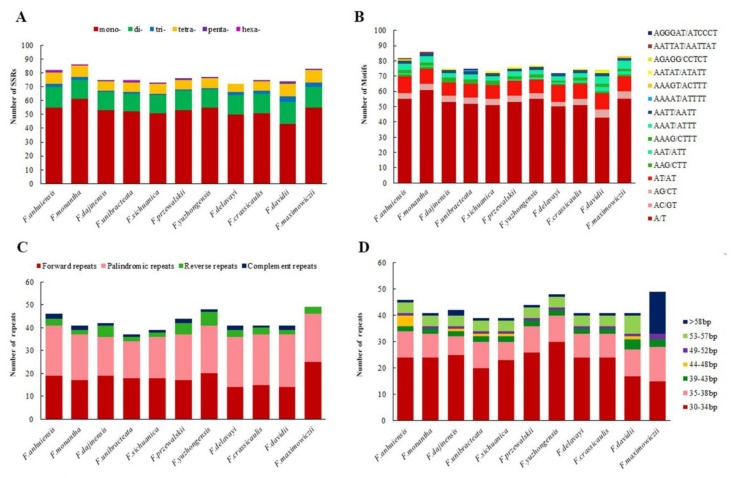
Analysis of different repeats in eleven chloroplast genomes of *Fritillaria*. (**A**) Number of different simple sequence repeat (SSR) types detected; (**B**) total numbers of different SSR motifs; (**C**) the number of four repeats types; (**D**) frequency of repeats by length.

With MISA analysis, a total of 848 SSRs were identified across the 11 newly sequencing *Fritillaria* plastomes. Each plastome/species was found to contain the number of SSRs ranging from 72 (*F. delavayi*) to 86 (*F. monantha*), with 20 SSRs shared between all 11 plastomes (Table S5, Figure 4A). Six types of SSRs were detected and the overall length ranged from 10 to 23 bp (Figure 4A, Table S5). Mono- (all A/T), di- (mostly AT/AT and AG/CT), and tetra-nucleotide (AAAG/CTTT, AAAT/ATTT, and AATT/AATT) SSRs were present in each plastome. Tri-nucleotide (AAG/CTT) SSRs were present in nine plastomes except for *F. przewalskii* and *F. yuzhongensis*, and (AAT/ATT) SSRs were present in eight plastomes except for *F. dajinensis*, *F. unibracteata*, and *F. sichuanica*. Penta-nucleotide (AATAT/ATATT) SSRs were in seven plastomes except for *F. monantha*, *F. unibracteata*, *F. delavayi*, and *F. maximowiczii*. By contrast, hexa-nucleotides (AATTAT/AATTAT) were only found in *F. anhuiensis* and *F. monantha* and penta- (AAAAT/ATTTT) and hexa-nucleotides (AGGGAT/ATCCCT) were only in *F. unibracteata*. Furthermore, penta- (AGAGG/CCTCT) nucleotides were only found in *F. davidii*, and di- (AC/GT) and penta-nucleotides (AAAGT/ACTTT) were only in *F. maximowiczii* (Figure 4B). Overall, mono-nucleotide SSRs were the most abundant type (68.28%) appearing among all 848 SSRs (Figure 4A). In the total 848 SSRs, most loci were situated in the intergenic spacer (IGS) regions, but some were found in *rpoC2*, *rpoB*, *accD*, *cemA*, *rpl22*, *ycf2*, *ndhD*, *ndhG*, *ndhH*, and *ycf1*coding genes (Table S5). Moreover, most of the SSR loci were situated in the LSC region (75.8%), followed by the SSC region (19.2%) and a minimum in the IR regions (5.0%) (Table S5). In addition, we found 41 polymorphic SSRs between the eleven species of *Fritillaria* (excluding mononucleotide SSRs) (Table S6), which could be useful for further population genetic studies.

We identified a total of 471 repeats including 196 forward, 224 palindromic, 36 reverse, and 15 complement in the eleven newly sequencing *Fritillaria* plastomes using REPUTER (Table S7). *F. maximowiczii* and *F. yuzhongensis* possessed the greatest total number of repeats (49 and 48), while *F. unibracteata* and *F. sichuanica* contained the fewest (39) (Figure 4C). Each *Fritillaria* plastid genome contained the number of large repeat sequences ranging from 30 to 57 and repeats with highest proportion ranging in size between 30 and 39 bp (Figure 4D). Repeats situated in homologous regions with identical lengths were recognized as shared repeats [13,26]. Under this criterion, there were 18 repeats shared by all eleven *Fritillaria* species, 24 repeats shared by ten of the *Fritillaria* species (except for *F. maximowiczii*), as well as 27 repeats shared by nine of the *Fritillaria* species (except for *F. maximowiczii* and *F. davidii*). Additionally, *F. maximowiczii* owned the most unique repeats (23), whereas four species had no unique repeats, namely *F. monantha*, *F. sichuanica*, *F. przewalskii*, and *F. crassicaulis* (Table S7).

### 2.4. Phylogenetic Analyses

**Figure 5:**
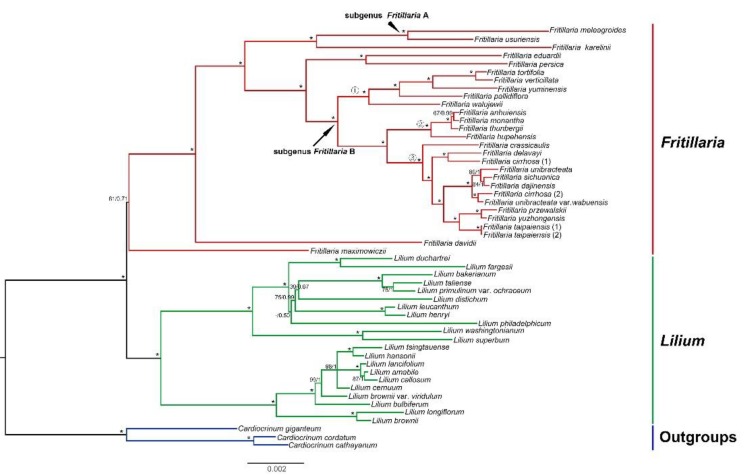
Phylogenetic relationship of the 52 species inferred from maximum likelihood (ML) and Bayesian inference (BI) analyses based on 64 shared protein-coding genes. Support values marker above the branches follow the order BS (bootstrap support)/pp (posterior probability), and “*” indicates 100% support values in both ML and BI trees. Accessions from different genera are written using different colors. ①, ②, and ③ indicate *F. pallidiflora*, *F. thunbergii*, and *F. cirrhosa* subclades, respectively.

Both Bayesian inference (BI) and maximum likelihood (ML) methods based on the 64 common CDS shared among the 52 plastomes (28 *Fritillaria*, 21 *Lilium*, and 3 *Cardiocrinum* species) generated almost identical topologies (Figure 5). *Lilium* was strongly supported as a monophyletic group (ML bootstrap support, BS = 100%, posterior probability, PP = 1). However, the monophyly of *Fritillaria* was moderately supported (BS = 81%, PP = 0.71). Within the Old World clade of *Fritillaria*, relationships between five subgenera (except subgenus *Korolkowia* and *Japonica*) were fully supported with generally high support values. The phylogenetic trees in this study also indicated subgenus *Fritillaria* was not monophyletic which divided into two clades, and *Fritillaria* clade B formed three monophyletic subclades (*F. pallidiflora*, *F. thunbergii,* and *F. cirrhosa* subclades). This was in accordance with our previous phylogenetic analysis based on three plastid markers [17]. Within *Lilium*, two strongly supported lineages formed the backbone of the phylogeny. However, the monophyly of four sections (section *Sinomartagon*, *Martagon*, *Leucolirion*, and *Pseudolirium*) were not supported based on our limited samples. In addition, the topologies from the single-copy genes and the whole complete chloroplast genome sequences were similar to that from the CDS sequences, and most lineages possess high bootstrap values. Rather unexpectedly, however, the monophyly of *Fritillaria* was weakly supported (BS = 67%, PP = 0.52) based on 64 single-copy genes (Figure S3) and not recovered based on whole genome sequences (Figure S4).

## 3. Discussion

### 3.1. Comparison of Fritillaria Plastomes and Phylogenetically Informative Markers

Our results revealed that the whole plastome sequences of 11 newly sequenced *Fritillaria* species were conserved not only in genome size, but also in gene content, structure, and order (Figure 1), which was consistent with previously published *Fritillaria* plastomes studies [23,24,25]. Nevertheless, some CDS genes exhibited high variations, mainly in some pseudogenes, for example *ψycf15*, *ψycf68*, and *ψinfA*, which were also reported in other angiosperm plastid genomes [7,9,26,27]. It is worth noting that there are only *F. crassicaulis*, *F. dajinensi*s, *F. delavayi*, *F. sichuanica*, and *F. unibracteata* having *ψycf15* and *ψycf68* in our results, indicating a few common aspects in their evolutionary processes and functions. The absence or presence of *ψycf15* and *ψycf68* in other *Fritillaria* species has also been reported by Li et al. [25]. The same phenomenon has also been observed in *Ipomoea* [28]. Additionally, the functions of pseudogene *ycf68* and *ycf15* are equivocal in all kinds of land plants [28], for example, in Podophylloideae, the *ycf15* gene was detected as non-functional because of the presence of an interrupted sequence where large amounts of stop codons were located [2]. The *infA* gene, which involved in codes of translation initiation factor 1 and an assembly of the translation initiation complex [29,30], was detected as a pseudogene in seven *Fritillaria* plastomes while completely lost in other four *Fritillaria* plastomes (Table S1). Similar phenomena have been also observed in other angiosperm plastid genomes, for instance, those of *Primula* species, only the plastid genome of *Primula poissonii* contains the *ψinfA* [31]. Our study indicates that the pseudogenization of *infA* may be a synapomorphy for the *F. cirrhosa* subclade.

Plastomes sequenced in our study are also largely similar in overall gene content and structure when compared with some previously published plastomes in Liliales [9,26,32,33,34,35,36,37]. However, the LSC-IR borders of Smilacaceae, Melanthiaceae (*Paris*), and Campynemataceae vary from those of *Fritillaria* (and other Liliales) by showing expansions (Figure S1), which may assist to stabilize the structure of the entire plastome as well as the prevention of gene gain and gene loss phenomenon [2,38,39,40]. By contrast, the SSC-IR boundaries of Melanthiaceae (*Veratrum*) feature contractions when compared to *Fritillaria* and other Liliales (Figure S1). Our results included 6 out of 10 families in the order Liliales (Table S2) and are in accordance with those of Do et al. [33], who used four families in Liliaeles and Do et al. [41], and five families in Liliales. Wang et al. [42] suggested that *trnH-rps19* clusters were located in the IR/LSC junctions within Liliales taxa, but different patterns of junction were found in Liliales from the present study (Figure S1). Furthermore, more studies covering all of 10 families should be performed to investigate the whole evolutionary trends of plastomes in Liliales. In general, the contraction and expansion of IR regions are relatively common evolutionary events in plants and have been used as evolutionary loci for phylogenetic relationships [1,31,43,44,45]. Li et al. reported that IR/LSC junctions expanded into *rps19* and suggested the events seem to be an ancestral symplesiomorphy of Liliaceae [26]. Here, we also found the feature but there was no obvious phylogenetic implication and further evidence was needed using sufficient genera of Liliaceae.

The cpDNA sequences from a variety of intergenic spacers (*trnL*-*trnF*) and genes (*matK*, *rbcL*, *rpl16*, and *atpB*) have often been used to infer the phylogeny of genus *Fritillaria* [16,17,46,47,48]. From our results, these frequently used plastid barcodes, such as *atpB*, *rpl16*, and *rbcL*, are among the relatively least informative genes (Figure 3). Therefore, the previous phylogenetic analysis based on these barcodes generated low resolution, especially for deep phylogenetic relationships with short internodes and fast rates, such as subgenus *Fritillaria*. By contrast, our sliding window analyses find six intergenic spacer regions with the highest Pi (>0.02) values from 26 *Fritillaria* species (Figure 3). We suggest these hotspot regions are valuable loci to understand the phylogenetic relationships for *Fritillaria* lineages (subgeneric levels) which have experienced rapid radiation.

### 3.2. Phylogenetic Relationships and Implications

Phylogenetic analyses based on 64 protein-coding genes generated a well resolved tree (Figure 5). Genus *Lilium* was sister to *Fritillaria*, and the monophyly of *Fritillaria* was recovered although moderate support values were detected. These results were different from a previous study, which showed that genus *Fritillaira* was paraphyletic and *Lilium* was nested within *Fritillaria* with moderate support values [17]. This difference may be attributed to our smaller sampling size, especially only one sample (*Fritillaria maximowiczii*) in the New World Clade (subgenus *Liliorhiza*). However, the phylogenetic results from single-copy genes and the whole chloroplast genomes showed that the monophyly of *Fritillaria* was weakly supported (BS = 67%, PP = 0.52) (Figure S3) and not recovered (Figure S4). Therefore, the relationships between the New World and the Old World clades of *Fritillaria* and *Lilium* still remain unsolved. In comparison with previous studies [24,25], our results detected further phylogenetic relationships between *Fritillaria* and *Lilium* with more plastomes data, and the subgenus *Fritillaria* was confirmed polyphyletic, which has a high resolution than previous studies (Figure 5) [16,17]. Our results also proved that the plastome data are of great advantages in phylogenetic analyses, although more extensive plastid genomic sampling was needed for further resolution of *Fritillaria* and *Lilium*.

## 4. Materials and Methods

### 4.1. Taxon Sampling, DNA Extraction, and Sequencing

We generated new plastome data for 11 species of *Fritillaria*. Fresh leaves of each species were collected in the field and dried with silica gel. All samples were obtained in China and field sampling was permitted by Natural Reserves in the Heilongjiang, Sichuan, Yunnan, Gansu, Anhui, and Zhejiang provinces. The vouchers were identified by Dr. Jiao Huang and were deposited in the Herbarium of Sichuan University (SZ); the voucher information is presented in Table S8. Total genomic DNAs were extracted from leaf material following the protocols using a plant genomic DNA kit (Tiangen Biotech, Beijing, China). The isolated genomic DNA was used to generate average 350 bp paired-end (PE) library according to the Illumina Hiseq platform (Illumina, San Diego, CA, USA), and sequenced by an Illumina genome analyser (Hiseq PE150).

### 4.2. Chloroplast Genome Assembly, Annotation, and Structural Analyses

FastQC v0.11.7 was used to assess the quality of sequenced raw reads [49]. Then, we sieved the chloroplast genome related reads by mapping all raw reads to the chloroplast genome sequences downloaded from NCBI of the genera *Fritillaria* and *Lilium* (38 species). Contigs, assembled from all related reads using SOAPdenovo2 [50], were sorted and joined into a single-draft sequence with *Fritillaria cirrhosa* (KF769143), *Fritillaria thunbergii* (NC034368), *Fritillaria taipaiense* (KC543997), and *Lilium leucanhum* (NC035590) as reference species in the software Geneious v11.0.4 [51]. Some gaps in the assembled plastomes were corrected using Sanger sequencing. The primers were designed by Primer 5.0 (Premier, Palo Alto, CA, USA). Primer synthesis and the sequencing of the polymerase chain reaction products were performed by Sangon Biotech (Shanghai, China). The amplifications and primers are shown in Table S9. Annotations of the complete plastomes were conducted using Geneious v11.0.4. The draft annotation was checked and edited manually following the reference genome to accurately confirm the start/stop codons and the exon/intron borders of genes. Furthermore, the schematic diagram of the circular plastome map was generated utilizing OGDRAW [52]. Eleven newly sequenced plastomes have been deposited in GenBank (accession numbers: MK258138-MK258148). The gene order and structure of 11 *Fritillaria* plastomes were compared using Geneious v11.0.4. We used the plastome of *Fritillaira crassicaulis* as a representation to further compare with plastomes of other 10 Liliales species (see Table S2).

### 4.3. Genome Comparative Analysis and Identification of Hypervariable Regions

Chloroplast genome comparisons across the eleven *Fritillaria* species was performed by the mVISTA program [53], using *F. cirrhosa* (KF769143) as the reference. For identifying hypervariable regions in *Fritillaria* and facilitating its utilization for future genetic population and species identification studies, multiple sequence alignments of 26 *Fritillaria* species (15 species were downloaded from NCBI; Table S10) were performed in MAFFT v.7 [54], and the software MEGA v.6 was used to adjust manually where necessary [55]. A sliding window analysis in DNASP v5.10 [56] was conducted for the sequence alignment to evaluate nucleotide diversity (Pi) including all protein-coding and noncoding (intron and intergenic spacer) regions. The extraction was made under the following two criteria: (a) mutation site >0; and (b) an aligned length >200 bp. Neighbor-Joining (NJ) trees were constructed by hypervariable markers in the noncoding regions using MEGA v.6 based on a k2Pdistance model.

### 4.4. Characterization of SSRs and Repeat Sequences

MISA perl script [57] was used to detect simple sequence repeat (SSR) loci of the 11 *Fritillaria* plastid genomes. The minimum numbers (thresholds) of repeats were 10, 5, 4, 3, 3, and 3 for mononucleotide, dinucleotides, trinucleotides, tetranucleotides, pentanucleotides, and hexanucleotides, respectively. The size and position of repeat sequences are assessed by REPuter [58], including inverted (palindromic), direct (forward), reverse, and complement repeats. The following constraint sets for repeat identification were used: (1) 90% greater sequence identity; (2) hamming distance equal to 3; and (3) a minimum repeat size of 30 bp.

### 4.5. Phylogenetic Analyses

Phylogenetic analyses were performed for the 28 *Fritillaria* species (11 species sequenced here) and 21 *Lilium* species, using three *Cardiocrinum* species as outgroups based on previous studies [17] (Table S10). Two accessions were included for *F. cirrhosa* and *F. taipaiense*. The analysis was performed based on an alignment of 64 protein-coding genes from the plastid genomes of the 52 species. The sequences of the 64 common CDS were extracted and aligned using MAFFT v.7 [54]. MEGA v.6 was used to adjust manually where necessary [55]. Topologies were constructed using both Bayesian inference (BI) and maximum likelihood (ML) methods. We used the Akaike Information Criterion (AIC) in JModeltest v.2.1.7 to determine the best-fitting models of nucleotide substitutions [59]. The GTR+I+G models were most suitable for both datasets. ML analyses were performed using RAxML-HPC2 [60] on XSEDE of the CIPRES Science Gateway [61], and statistical node supports were estimated via a bootstrap analysis. Bayesian inference (BI) analyses were conducted in MrBayes v3.2 [62]. Two independent Markov Chain Monte Carlo chains were run simultaneously for five million generations. The trees were sampled every 100 generations with the first 25% of calculated trees discarded as burn-in. The consensus tree was constructed from the remaining trees to estimate posterior probabilities (PPs). Additionally, the complete chloroplast genome sequences and 52 extracted single-copy genes were also collected to perform the phylogenetic analyses, respectively.

## 5. Conclusions

Our study is the first to report the eleven whole plastomes of *Fritillaria* of which the organization is described. The comparison of these plastid genomes among each other as well as with other species of Liliales showed high similarities in entire structure and content. The synteny of gene order for eleven *Fritillaria* plastomes is also rather conserved. However, these plastid genomes show some structural variations at the junctions of their four regions due to the expansion or contraction of the IRs, especially obvious in Liliales. The six noncoding-cpDNA regions are identified as the fastest evolving loci from the comparison of DNA sequence divergence among 26 *Fritillaria* plastomes. Therefore, these highly variable loci, and 471 repeat sequences from eleven *Fritillaria* plastoms can be used for future phylogenetic and phylogeographic analysis. Furthermore, the plastid genomes SSRs with rich diversity identified herein could be useful for further population genetic studies of *Fritillaria*. 

This study performed a new level of phylogenomic sampling, and found that *Fritillaria* was monophyletic and sister to *Lilium*. Future phylogenomic studies require more extensive taxonomic sampling, especially subgenus *Liliorhiza* of genus *Fritillaria* to discern the relationships among the Old and New World clades of *Fritillaria* and *Lilium*. Moreover, some efforts should focus on the evolutionary and adaptive researches of *Fritillaria* species in the future as the recent studies suggested [63,64]. In conclusion, our results will be valuable to understand the evolutionary relationship between *Fritillaria* and *Lilium*, especially plastid gene evolution of *Fritillaria*.

## Figures and Tables

**Table 1:** Summary of major characteristics of eleven *Fritillaria* plastomes, including aspects of genome size, G-C content, and gene number (per type and location).

Species	Genome Size	LSC Length	IR Length	SSC Length	G-C	Number of Genes				Number of CDS			
	(bp)	(bp)	(bp)	(bp)	(%)	Total	CDS	rRNAs	tRNAs	LSC	IR_A_	SSC	IR_B_
*F. anhuiensis*	152,196	81,950	26,340	17,566	36.9	131[18]	85[6]	8[4]	38[8]	60	7	11	7
*F. crassicaulis*	151,852	81,615	26,345	17,547	37.0	136[20]	90[8]	8[4]	38[8]	60(1)	9(3)	11	9(2)
*F. dajinensis*	151,971	81,728	26,351	17,541	37.0	136[20]	90[8]	8[4]	38[8]	60(1)	9(3)	11	9(2)
*F. davidii*	152,044	81,896	26,232	17,684	37.0	131[18]	85[6]	8[4]	38[8]	60	7	11	7
*F. delavayi*	151,948	81,683	26,357	17,551	36.9	136[20]	90[8]	8[4]	38[8]	60(1)	9(3)	11	9(2)
*F. maximowiczii*	152,434	81,976	26,574	17,310	37.1	132[18]	86[6]	8[4]	38[8]	60(1)	7	11	7
*F. monantha*	152,158	81,895	26,350	17,563	37.0	131[18]	85[6]	8[4]	38[8]	60	7	11	7
*F. przewalskii*	151,688	81,448	26,352	17,536	37.0	132[18]	86[6]	8[4]	38[8]	60(1)	7(1)	11	7
*F. sichuanica*	151,967	81,727	26,350	17,540	37.0	136[20]	90[8]	8[4]	38[8]	60(1)	9(3)	11	9(2)
*F. unibracteata*	151,971	81,730	26,350	17,541	36.9	136[20]	90[8]	8[4]	38[8]	60(1)	9(3)	11	9(2)
*F. yuzhongensis*	151,652	81,424	26,351	17,526	37.0	132[18]	86[6]	8[4]	38[8]	60(1)	7(1)	11	7

Abbreviations: CDS, protein-coding sequences/genes; LSC, large single-copy region; SSC, small single-copy region; IR, inverted repeat (A or B) regions. Numbers in brackets mean the number of duplicated genes, e.g., 131[18] means there were 131 total genes in the plastome of which 18 were duplicated in the IRs. Numbers in parentheses mean the number of pseudogenes, e.g., 61(1) means there were 61 unique genes observed in LSC of which one was pseudogene.

## Supplementary Materials

The following are available online at https://www.mdpi.com/2223-7747/9/2/133/s1, Table S1: Gene composition in the eleven *Fritillaria* plastid genomes. Table S2: Summary of accession numbers and genome sizes in Liliales. Table S3: Comparison of nucleotide diversity (Pi) values among protein-coding genes (CDS) in 26 *Fritillaria* plastomes. Table S4: Comparison of nucleotide diversity (Pi) values among intergenic spacer (IGS) and intron of 26 *Fritillaria* plastomes. Table S5: Statistics of simple sequence repeats in each species. Table S6: Simple sequence repeat (SSR) polymorphism in eleven *Fritillaria* chloroplast genomes. Table S7: Analyses of repeat sequences in eleven *Fritillaria* chloroplast genomes. Table S8: Voucher information for species newly sequenced for this study, including collector’s name and collection number, geographical locality and herbarium acronym where the specimen is deposited. Table S9: Primers used for gap closure in this study. Table S10: Accession numbers of chloroplast genome sequences included in phylogenetic analyses. Figure S1: Comparison of LSC, IRs, SSC junction positions and gene loss among Liliales plastomes. Figure S2: Sequence identity plots between eleven *Fritillaria* chloroplast genomes, with *F. cirrhosa* as a reference. Annotated genes are displayed along the top. The vertical scale represents the percent identity between 50 and 100%. Genome regions are color coded as protein coding, rRNA coding, tRNA coding, or conserved non-coding sequences (CNS). Figure S3: Phylogenetic relationship of the 52 species inferred from ML and BI analyses based on 64 shared single-copy genes. Support values marker above the branches follow the order BS (bootstrap support)/pp (posterior probability), and “*” indicates 100% support values in both ML and BI trees. Accessions from different genera are written using different colors. ①, ②, and ③ indicate *F. pallidiflora*, *F. thunbergii*, and *F. cirrhosa* subclades, respectively. Figure S4: Phylogenetic relationship of the 52 species inferred from ML and BI analyses based on whole genome sequences. Support values marker above the branches follow the order BS (bootstrap support)/pp (posterior probability), and “*” indicates 100% support values in both ML and BI trees. Accessions from different genera are written using different colors. ①, ②, and ③indicate *F. pallidiflora*, *F. thunbergii*, and *F. cirrhosa* subclades, respectively.
