# Supplementary figures and images for: Comparative Chloroplast Genomics of Fritillaria (Liliaceae), Inferences for Phylogenetic Relationships between Fritillaria and Lilium and Plastome Evolution
Source: Plants (Basel). 2020 Jan 21;9(2):133. doi: 10.3390/plants9020133 (PMC7076684; doi:10.3390/plants9020133)

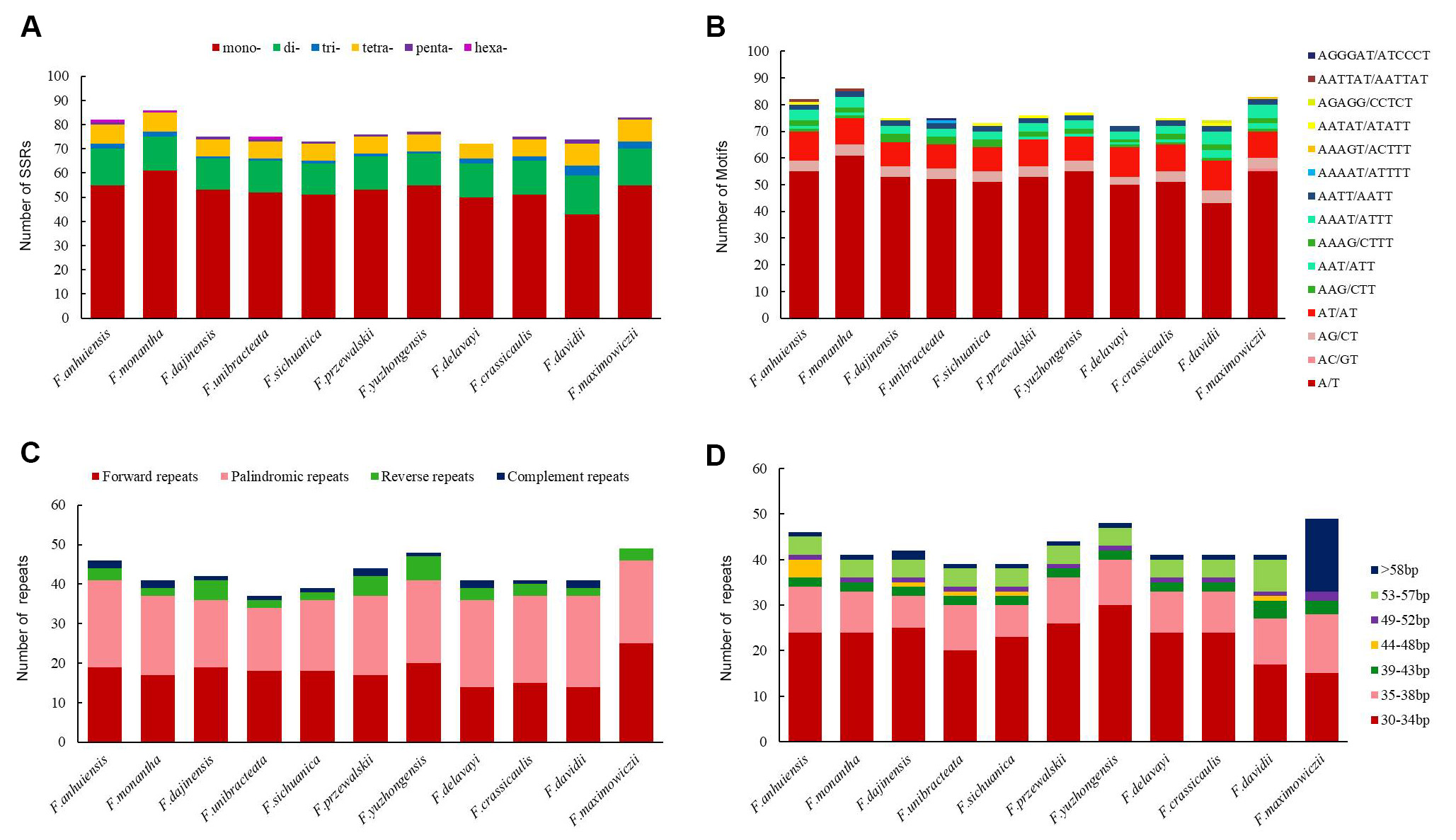

Supplement: Supplementary file 1 [file plants-09-00133-s001.zip › fig.4 and fig.5/Fig.4-gai.jpg]

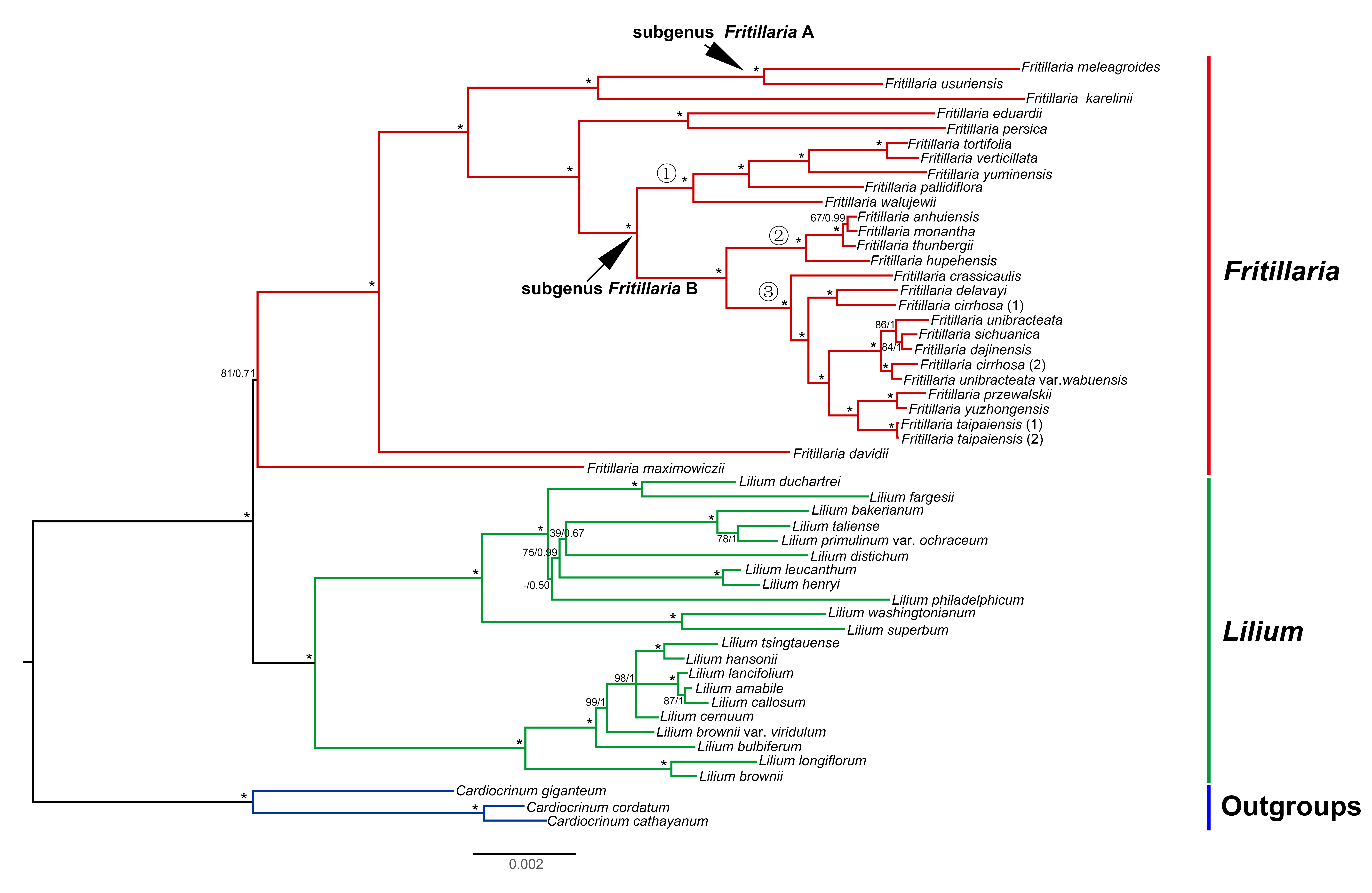

Supplement: Supplementary file 1 [file plants-09-00133-s001.zip › fig.4 and fig.5/Fig.5-gai.jpg]
